# The ETHICA-4P in intercultural mental health care for migrants: perspectives from the United Kingdom and Chile

**DOI:** 10.1080/16549716.2026.2758969

**Published:** 2026-11-04

**Authors:** Clara Calia, Benny Martin, Liz Grant, Cristobal Guerra, Edgardo Toro, Magda Liliana Rincón, Alice Gritti, Corinne Reid

**Affiliations:** a The School of Health in Social Science, The University of Edinburgh, Edinburgh, UK; b Global Health Academy, The University of Edinburgh, Edinburgh, UK; c Escuela de Psicología, Facultad de Ciencias Sociales, Universidad Santo Tomás, Viña del Mar, Chile; d Escuela de Trabajo Social, Pontificia Universidad Católica de Valparaíso, Valparaiso, Chile; e Facultad de Bioética, Universidad Anáhuac, México

**Keywords:** Ethical decision-making, cultural humility, reflexive practice, clinical ethics, migrants

## Abstract

**Background:**

Mental health services in destination countries for migration are increasingly required to support service users from diverse cultural backgrounds. Although migrants may experience elevated mental health challenges, existing approaches are often insufficient to meet their needs. There is a need for tools that support practitioners in delivering competent and ethical intercultural care and navigating complex ethical decision-making.

**Objective:**

To beta-test the ETHICA-4P ethical decision-making toolkit and evaluate its potential to support clinicians in addressing ethical challenges arising in intercultural practice.

**Methods:**

Feedback was collected from stakeholders and practitioners in two destination countries for migration, Chile and the United Kingdom, through focus groups and anonymous surveys. Data were analysed using Reflexive Thematic Analysis to compare participants’ perspectives and suggestions regarding the use and further development of the toolkit.

**Results:**

Three themes were identified: ‘Shared ethical dilemmas’, ‘Prioritising different levels of analysis according to place-based needs’, and ‘Practical guide, or tool for reflection?’. Participants reported similar ethical challenges and shared perspectives on the potential use of ETHICA-4P in practice. However, they differed in how intercultural care needs were conceptualised and addressed according to context, with some emphasising systems-level responses and others clinician-level approaches. These differences generated distinct suggestions for improving the toolkit.

**Conclusions:**

The findings provide guidance for the continued development of ETHICA-4P and highlight the importance of flexibility in supporting ethical intercultural practice across diverse service and national contexts.

## Paper Context

- **Main findings:** Participants in two national contexts identified shared ethical dilemmas in intercultural mental healthcare, and the toolkit showed potential to support reflective practice.
- **Added knowledge:** The study provides contextual insights into how practitioners may use an ethical decision-making toolkit when working across cultures.
- **Global health impact for policy and action:** Findings support the development of culturally and structurally responsive ethics resources for equitable mental health services.

## Background

Globally, over 281 million people live outside their country of origin, including nearly 46 million forcibly displaced individuals [1]. Recent migration has increased cultural diversity in destination countries [2,3] and mental health services must increasingly support service users from diverse cultural backgrounds [4]. Migrants, particularly those displaced forcibly or in childhood, have a higher risk of mental health challenges [5–7]. A number of barriers have been identified to accessing mental health services including social, economic, cultural and communication barriers [8], along with fear and mistrust of authorities, shame, and discomfort disclosing sensitive issues [9] Migrants often underuse mental health services or receive inadequate care [10–12]. The WHO calls for services that are culturally appropriate and responsive [13].

Providing adequate and equitable intercultural care is required to meet core biomedical ethical principles [14] but remains challenging. Ethical clinical decision-making is complex when clients’ beliefs, frameworks, and expectations differ from those of practitioners [15]. There is a recognised need for tools to support clinicians in better navigating these challenges [16,17].

This study tests the potential of ETHICA-4P, an Ethics Toolkit for Harnessing Integrity in Complex Arenas, to support practitioners working interculturally in mental health care. It draws on perspectives from participant groups in the United Kingdom and Chile, two distinct contexts for intercultural mental healthcare. Small scale beta testing is especially important in product design in contexts where ‘doing no harm’ is a challenging priority in service design and delivery – in mental healthcare it is vital to ensure that development, design, usability, accessibility and embedded evaluation processes are fit for purpose and that bugs are ironed out before widescale toolkit launch.

## Ethics, cross-cultural and intercultural complexity

Ethics concerns moral principles guiding behaviour. Mental health clinicians support vulnerable people and must design effective intervention with consideration of ethical responsibility [16,18]. Codes and laws provide frameworks but do not replace situational judgment; ethical decision-making requires active reflection [18,19]. ‘Intercultural’ is used here to emphasise respectful interactions between cultures, working collaboratively together, in the creation and negotiation of shared understandings; in contrast, ‘cross-cultural’, focuses on comparison and recognising differences in experience and ways of working in different places [20]. This study explores perspectives arising from participant groups in Chilean and United Kingdom contexts, recognising that differences in mental health service provision and other contextual factors may shape how participants engage with, and perceive the utility of, the toolkit. In both cases, we are interested in how the ETHICA-4P toolkit may (or may not) support ethical intercultural practice when mental health practitioners are working with migrants from other cultures, where the purpose is to create shared understanding of need, and a shared platform for addressing mental health concerns.

Cultural difference adds complexity to ethical clinical decision-making. Culture involves shared understandings, practices, and values that are dynamic and shaped by social environments [21,22]. In practice, cultural identity is as individual as each person [23]. Distress is expressed and interpreted differently across cultures [15]. Global mental health frameworks, developed mainly in WEIRD contexts [24], may not fit all populations [25], thus affecting care outcomes [26]. Western models may unintentionally pathologise normal (yet unfamiliar) cultural phenomena [26]. An ethical approach to mental health must integrate cultural and structural awareness [27].

## Migration and intercultural mental health care in the United Kingdom

In 2022, 16% of the United Kingdom population was foreign-born. The United Kingdom also receives asylum seekers from diverse regions [28,29]. Mental health care is provided by the National Health Service (NHS) and private sector. The NHS Race and Health Observatory recognises disparities in care despite commitments to address them [30], though access can be limited for those without official status [31].

Practitioners are expected to consider culture, equality, and diversity [32]. However, cultural minorities underuse services, citing stigma, language barriers, and cultural insensitivity [33]. Cultural competency training varies in quality and has been criticised as inadequate [34–37].

## Migration and intercultural mental health care in Chile

Since 1990 Chile has become a major destination for regional migrants. The foreign-born population grew from 0.7% in 1982 to 7.5% in 2020 [38]. Although most migrants speak Spanish, dialect and language barriers still pose challenges [39]. Haitian Creole speakers, in particular, face difficulties highlighted by the case of Joane Florvil, who died following a failure of cultural understanding [40].

Public mental health care is accessible regardless of status, yet disparities persist [41]. Chile pioneered intercultural health initiatives for Indigenous groups but did not extend these to migrants [42]. Current policy acknowledges the need for such approaches.

## The ETHICA-4P

The Ethics Toolkit for Harnessing Integrity in Complex Arenas (ETHICA-4P) (Figure 1) was initially developed by four of the authors of the current paper who once worked together in Edinburgh but who hail from four countries and speak three languages. We shared our challenges of working interculturally in both research and clinical work, and expanded these conversations to include other colleagues in the global academic village. ETHICA-4P emerged through consultations with over 400 professionals from 40+ countries [43,44] and is designed to support clinicians navigating ethical challenges in complex, cross-cultural and intercultural settings. Available online and as a pocket guide in 24 languages, the toolkit is not positioned as a repository of facts but rather promotes reflection across ten stages of clinical contact.

**Figure 1.**
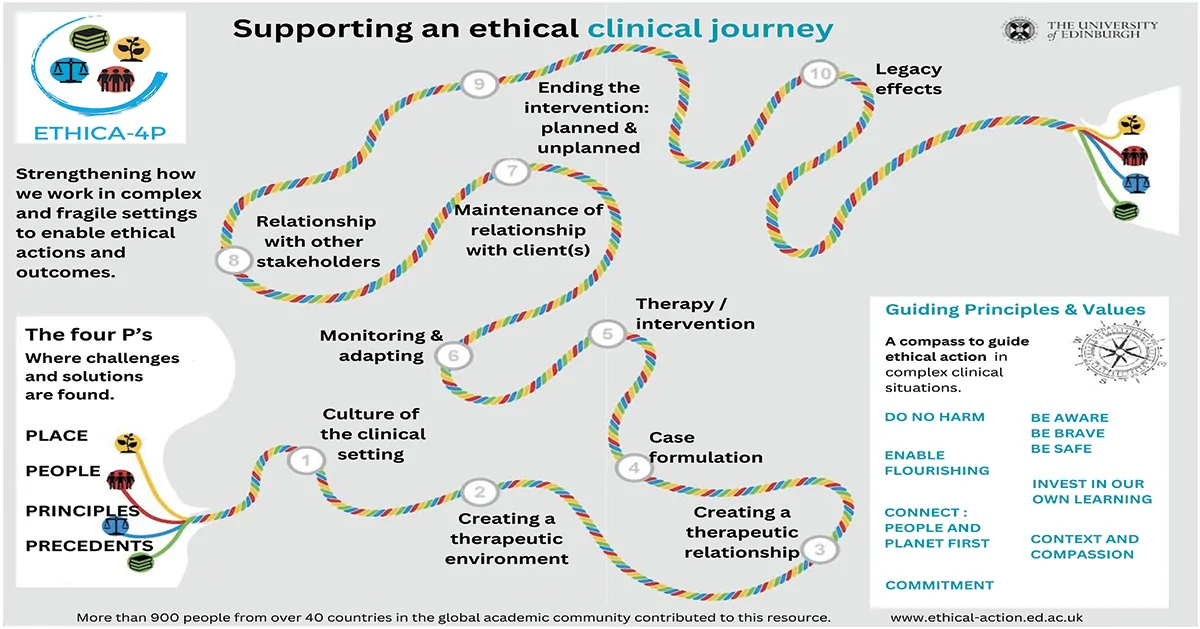
ETHICA-4P clinical toolkit.

It structures ethical consideration around four reflective threads:

- **Place**: Directing attention to cultural and structural contexts that may influence care needs and potential outcomes, including language, politics, and systems.
- **People**: The network of those involved in care foregrounding relationality, positionality, co-construction, and intersubjectivity.
- **Principles**: Core ethical values and potential risks that may influence mental healthcare; and supporting ethical deliberation concerning competing values and risks.
- **Precedent**: Past approaches and practices (successful and unsuccessful) that can inform decision-making; encouraging practitioners to draw critically on existing cultural, clinical, legal, and experiential knowledge without treating it as universally applicable.

The toolkit encourages iterative reflection as clinicians and service providers move through different stages of care. It supports movement between contextual knowledge, structural analysis, practitioner reflexivity, and collaborative meaning-making.

As part of a beta testing strategy, the expanded ETHICA-4P design team undertaking this study seeks to further evaluate its potential and gather feedback on improvements made to date. For more details about the toolkit, please see Figure 1.

## Methods

### Overview of design

This beta testing study explored perspectives on ETHICA-4P through focus groups and surveys with mental health professionals and stakeholders in the United Kingdom and Chile. Data were collected during workshops in both countries, presented by members of the ETHICA-4P design team at the University of Edinburgh and Universidad Santo Tomás, respectively, and through surveys following a presentation at the University of Edinburgh’s Impact Festival [44,45]. The two data-collection modes were selected for pragmatic and conceptual reasons: focus groups enabled in-depth discussion and critical debate, while anonymous surveys provided a private route for individual feedback, particularly relevant because members of the research team were also involved in developing ETHICA-4P. Using both sources enabled examination of convergence and divergence across individual written accounts and interactive group discussion [46]. An inductive Reflexive Thematic Analysis (RTA), guided by Braun and Clarke [47], was used to analyse the qualitative data.

### Researchers

The workshops in Chile were conducted by academics Magda Liliana Rincon (PhDc), and Dr Edgardo Toro (PhD), and those in the United Kingdom by academics Dr Clara Calia (PhD, DClinPsy), Dr Alice Gritti (PhD), and Dr Corinne Reid (MPsych, PhD). Five of the authors are also clinical psychologists with experience in intercultural and cross-cultural clinical practice. The analysis was conducted by Edinburgh MSc student Benjamin Martin (MSc), supervised by the lead author Dr Clara Calia. Please refer to the supplementary material for detailed information on the COREQ (Consolidated Criteria for Reporting Qualitative Research) checklist used in this study.

All researchers involved in conducting the focus groups and surveys were experts in qualitative research, with several published studies in the field. The Chilean researchers and two of the United Kingdom researchers have professional experience and academic interest in the field of clinical ethics. There were a mix of male and female researchers in both Chile and the United Kingdom, with a male researcher conducting the analysis in the United Kingdom and working together with the female lead author on the manuscript.

### Participants

United Kingdom focus group participants were purposively invited for their expertise or interest in intercultural mental healthcare, through researchers’ professional networks, via email. Attendees at the Impact Festival included students, academics, and professionals, recruited through convenience sampling, via email and poster. Twelve people took part in the United Kingdom focus groups and 15 completed anonymous surveys. See Tables 1 and 2.

**Table 1.** United Kingdom focus groups (*n* = 12).

Profession	*n*	%
Clinical Psychologist (CP)	2	16.7
Trainee Clinical Psychologist (TCP)	1	8.3
Student (MSc) (ST)	2	16.7
Charity Worker (CW)	2	16.7
Academic/Lecturer (AC)	2	24.9
Public Health Worker (PHW)	1	8.3
Psychotherapist (PS)	1	8.3
Support Worker (SW)	1	8.3

**Table 2.** United Kingdom survey respondents (*n* = 15).

Profession	*n*	%
Clinical Psychologist (CP)	2	13.3
Student (MSc) (ST)	6	40.2
Academic/Lecturer (AC)	1	6.7
Public Health Worker (PHW)	1	6.7
Psychotherapist (PS)	1	6.7
Support Worker (SW)	1	6.7
Global Mental Health Worker (GW)	1	6.7
Mental Health Researcher (R)	1	6.7
Clinician (unspecified) (C)	1	6.7

In Chile, 25 participants were recruited via snowball sampling from the researchers’ professional networks, via email. All participated in focus groups and completed surveys. See Table 3.

**Table 3.** Chilean participants (in both focus groups and surveys) (*n* = 25).

Profession	n	%
Clinical Psychologist (CP)	18	72
Trainee Clinical Psychologist (TCP)	7	28

Participants in all groups in both countries received a participant information sheet (PIS) informing them about the concept of the toolkit, the researchers’ connection to it, and the purpose of the research. The PIS explained that feedback would be used to develop the toolkit further. No participants declined to participate in the research.

### Materials and procedure

Anonymous surveys were distributed on paper (Chile) and via Qualtrics (United Kingdom). The survey included open-ended questions inviting participants to describe ethical challenges in mental healthcare and provide feedback on ETHICA-4P; these free-text responses formed the survey data used in thematic analysis. Professional-background information was used descriptively. Focus groups addressed the same broad topics, allowing deeper discussion of the toolkit’s model and stages.

Chile hosted three workshops in Spanish at Universidad Santo Tomás. Each included a presentation, case analysis, survey completion and a 45-minute focus group in which critical analysis was encouraged; only participants and researchers were present. In the United Kingdom, a workshop at the University of Edinburgh presented the toolkit and introduced case templates, followed by three parallel focus groups that were minuted by MSc students. Surveys were sent to workshop participants after the event. The toolkit was also presented at the Impact Festival, where the survey was promoted by QR code and email.

### Data management and analysis

Open-ended survey responses and focus-group records were treated as two qualitative sources addressing overlapping questions about ethical challenges and the use of ETHICA-4P. They were analysed within one RTA rather than as separate quantitative and qualitative components: no survey scores were transformed or ‘qualitised’, and the study did not use a mixed-methods analytical design. Free-text survey responses contributed directly to coding alongside focus-group data, enabling patterns of convergence and divergence across individual written accounts and group discussion to be examined.

Chilean focus-group transcripts and free-text survey responses were translated from Spanish into English by a bilingual Chilean psychologist familiar with the local linguistic and cultural context. Translation and analysis proceeded iteratively. During initial coding and theme development, BM worked with the bilingual Chilean researcher/translator to compare English translations with the Spanish originals. Where culturally specific meanings or linguistic nuances required clarification, these were discussed collaboratively and the English wording was refined to preserve participants’ intended meanings. Coding was then conducted in English using an inductive approach. Codes were generated across both data sources, reviewed and refined iteratively, and developed into themes and subthemes. The wider research team subsequently reviewed the developing interpretation to support reflexive discussion and refinement of the final thematic structure.

A qualitative, exploratory approach was chosen to examine how the toolkit might support or impede ethical decision-making. A critical realist stance informed by social constructionism guided interpretation, recognising participants’ accounts as relating to material and structural conditions while also being shaped by cultural, professional and social meaning-making.

#### Ethics

Ethical approval was obtained from the University of Edinburgh (25 May 2022; reference 22.5.22) and Universidad Santo Tomás (5 July 2023; reference 23,136,643-2023), covering data collection, translation and joint analysis. All participants received study information in advance and provided written informed consent. Participation was voluntary, surveys were anonymous, and focus-group material was reported using participant codes rather than names. Discussions concerned participants’ professional perspectives rather than identifiable service-user data, and only participants and researchers were present during the workshops. Data were handled in accordance with the approved institutional procedures and confidentiality agreements; because of the sensitive nature of the material, raw transcripts and survey responses are not publicly available.

### Results

Workshop conversations guided by the ETHICA-4P framework generated discussion of intercultural mental health challenges across the stages of the clinical journey and the four Ps: Place, People, Principles and Precedents. RTA identified areas of convergence and divergence in perspectives expressed by participants in the United Kingdom and Chilean samples. These contextual perspectives informed shared and distinct suggestions for improving ETHICA-4P and illustrated how participants engaged with the framework in different ways. Through iterative and reflexive interpretation, three major themes were developed (Figure 2):

1. **Highlighting Shared Ethical Dilemmas** – common areas of ethical tension in intercultural mental health work.
2. **Prioritising Different Levels of Analysis According to Place-based Needs** – contrasting approaches to addressing these tensions according to intercultural context, and recommendations for the toolkit.
3. **Practical Guide or Space to Reflect?** – layered rich views on the toolkit’s purpose and use in practice.

**Figure 2.**
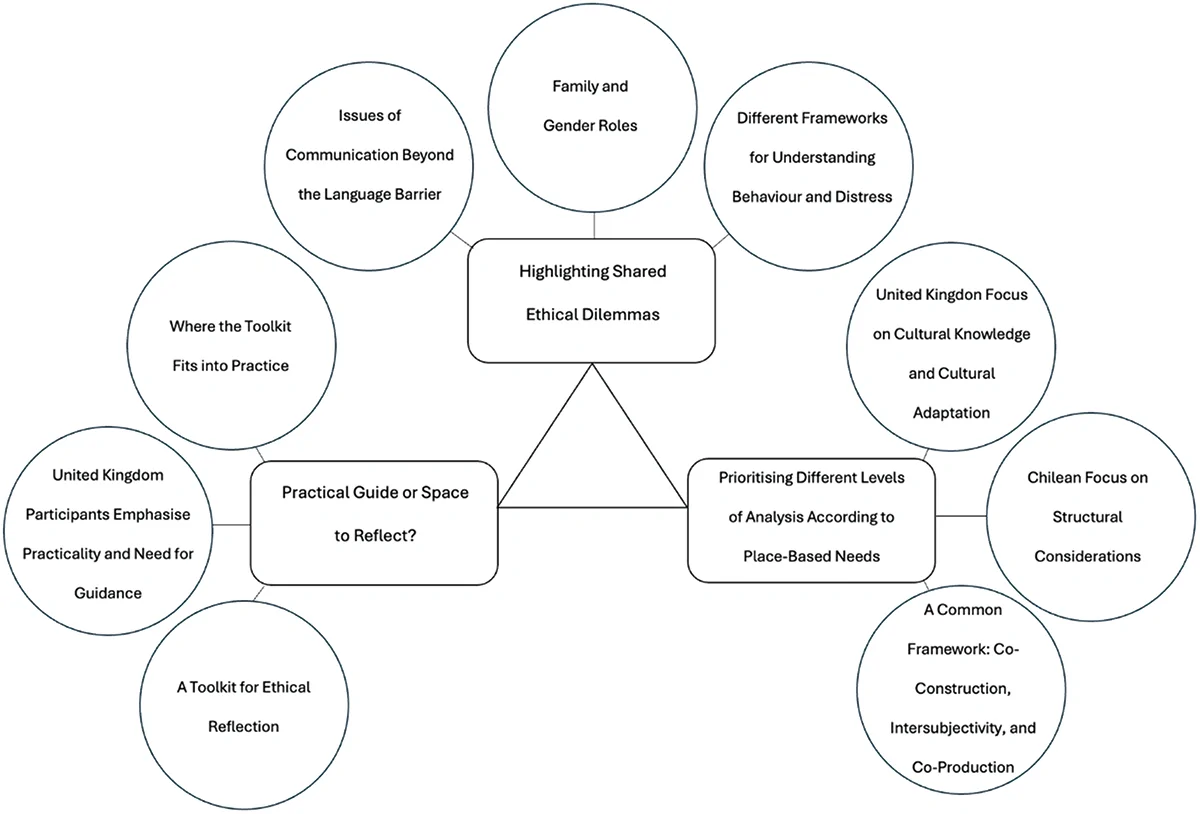
Map of themes and subthemes.

### Theme 1: highlighting shared ethical dilemmas in working interculturally

Consideration of the stages of the clinical journey through the lens of Place, People, Principles and Precedents (the 4P’s) generated ethical dilemmas that were shared across participants in both samples, suggesting the potential relevance of ETHICA-4P across these two distinct study contexts.

Notably, ethical dilemmas common to both countries and contexts when working inter-culturally, included:

#### Issues of communication beyond the language barrier

‘*Language barriers*’ (UKS15ST, ChileS1CP) were cited by both United Kingdom and Chilean participants as a significant issue which ‘*very often prevents people from even engaging in services*’ (UKS5R). However, they noted that this often represents ‘*a communication barrier, not only a language competency barrier*’ (UKFG11CW). In Chile, where most migrants share a common language, participants still reported misunderstandings because of different ‘*ways of speaking*’ (ChileFG6CP). This related to an acknowledgement that mental health language differs culturally as well as linguistically (UKFG11CW). A Chilean participant explained:

Sometimes when we say something we just assume someone else understands the same thing, like how we interpret emotions versus how they experience them in other cultures (ChileFG2CP)

It was recognised that interpretation can lead to ‘*violating intimacy/privacy and even losing a lot of information due to the biases of the translator*’ (ChileS20CP). Problems were identified with interpreters causing discomfort, sometimes because they come from the same small community as service users (UKFG1CW), which can amplify confidentiality and privacy concerns (UKS12S), particularly for certain groups; ‘*an LGBTQA+ person might not be comfortable with an interpreter from their own community, fearing stigmatisation or violence*’ (UKS4SW).

A lack of training was also noted: ‘*Very few translators have had training in mental health*’ (UKFG1CW). A Chilean participant illustrated this dilemma in a clinical context:

We had to involve someone who speaks Creole because otherwise the therapist couldn’t communicate … we created the most intimate, private setting, only to bring someone in who is not even a psychologist (ChileFG6CP)

Participants recognised that the toolkit mentioned language but thought it should be made ‘*more explicit*’ (ChileFG6CP) perhaps even part of the ‘5 Ps’: ‘*I was thinking of adding “P” for “Polyglot”, because I was thinking that language issues can be hidden, and it’s an important topic*’ (ChileFG2CP).

#### Family and gender roles

Participants identified recurrent ethical issues relating to ‘*gender and social norms*’ (UKS2TCP), ‘*gender violence*’ (ChileS3CP), ‘*Role conceptions (masculine, feminine, married, children)*’ (ChileS5CP), and violence towards children (ChileFG6CP). One United Kingdom participant found that:

Most issues I’ve faced have been concern around people’s attitudes to gender … This happened in a child development case where only the father would speak to me, despite the mother being in the next room. This may have been typical for that culture, but it raised an ethical issue with me around safeguarding. (UKS3CP)

Conversely, a Chilean participant reported that *‘in Venezuela there is a custom in which the man is provider and child-rearing is not shared*’, making this situation difficult to problematise (ChileFGTCP), another described experiences of working with families where gender violence had been normalised (ChileFG4CP), and it was highlighted that cultural norms around violence against children can persist at home even with legal protections in the public sphere: ‘*at school they may have told you that this is a violation of rights, but inside this house you are in Ecuador*’ (ChileFG6CP).

#### Different frameworks for understanding behaviour and distress

‘*Explanatory frameworks of mental wellness / distress*’ (UKS2GW) were cited as another challenge, engendering a situation where ‘*the foreigner’s experience is interpreted from a cultural reality that is not his or her own, which colours and influences the intervention*.’ (ChileS2CP). Participants problematised ill-fitting psychological models (UKS9C), leading to the imposition of understandings (UKS4SW, ChileFG2CP). One United Kingdom participant gave the example of:

Pathologisation of culturally normal behaviour for the service user … culturally normal understanding of magic, maraboutage or spirit/jinn possession, vs symptom of delusion. (UKS4SW)

Different expectations of the role of service provider and service user were also mentioned, in the form of different ‘*cultural understandings of the boundaries of professional relationships … leading to feelings of e.g. disappointment or abandonment*’ (UKS4SW).

### Theme 2: prioritising different levels of analysis according to place-based needs

One contextual contrast observed between the two participant groups concerned the level of service analysis that was foregrounded when using ETHICA-4P. Within this study, participants in the Chilean sample tended to foreground structural ethical challenges, whereas participants in the United Kingdom sample more often reflected on issues at the level of individual professional practice. Specifically:

#### Cultural knowledge and adaptation in the United Kingdom participant accounts

United Kingdom participants emphasised the importance of cultural ‘*knowledge*’ (UKS1PS, UKS4SW, UKS6CP) and cultural adaptation of treatment modalities (UKS1PS, UKS6CP, UKS9C).

They problematised ‘*gaps in knowledge*’ (UKS4SW) on the part of service providers and noted that it is difficult to understand someone’s culture ‘*if you are not informed*’ (UKS8ST). One participant wrote that a lack of knowledge can ‘*interfere in the therapy process*’ (UKS1PS) and suggested that the toolkit should provide or emphasise ‘*more education to the therapists on the different cultures they will be dealing with*’ (UKS1PS).

Participants also highlighted a need for culture-appropriate interventions to be developed (UKS1PS, UKS6CP), noting that current interventions such as CBT are ‘*westernised*’ and for individualist societies (UKFG1CW). It was proposed that the toolkit should cover ‘*how to adapt specific therapeutic techniques or assessment tools*’ (UKS9C).

#### Structural considerations in the Chilean participant accounts

In contrast, Chilean participants emphasised structural issues, highlighting the necessity of ‘*recognising the impact of migration trajectories, especially where multiple rights have been violated*’ to understand the structural causes of their service users’ distress (ChileS20CP). One participant argued that issues like domestic violence can be viewed in the context of the ‘uprooting’ of migration, rather than through a purely cultural lens:

One of the things that we must also make visible … is this uprooting, how we approach this as an expression of violence that they bring with them, that perhaps at the time of the intervention is more significant than any practices or experiences that may have occurred in their own countries (ChileFG4CP)

Participants discussed the difficulty of framing migrants’ experiences while operating from a sociopolitical context where ‘*lived experiences of harm are not common*’ (ChileS5R). A risk was identified that this could lead to practitioners ‘*relativising*’ experiences of harm ‘*narrated in contexts of vulnerability inherent to or accentuated by migratory processes*.’ (ChileS20CP). One participant described a clinical team’s difficulty formulating a case:

There were some who were astonished at the experiences he had at such a young age, while others regarded the experiences differently because their opinion was: “Well, that happens a lot in Colombia”. (ChileFG1CP)

Chilean participants valued the toolkit’s consideration of structural issues, with one suggesting that a major strength of the model lay in its ability to place practitioners in a ‘*transversal orientation*’ that cuts across many ‘*intersecting elements that relate to interculturality*’ (ChileFG6CP). However, they highlighted a need to understand ‘*legal conditions*’ in service users’ countries of origin (ChileFG4CP) and suggested that the toolkit could provide ‘*Information or training on the legal aspects of the countries involved*’ (ChileS17TCP).

#### A common framework: co-construction, intersubjectivity, and co-production

Chilean participants discussed co-construction and intersubjectivity in intercultural clinical work, seeing meaning as something created interactively by service provider and service user:

The way they see violence, their whole view of what family is, or what an institution is, is also different from ours. The idea is that in this therapy space we can co-construct what the idea of institution or of violence represents for us, and what it represents for them, and from there we will at least have a common or moderately common framework within which to work. (ChileFG15CP)

Participants pointed out that service users often ‘*find themselves being judged in relation to where they come from*’ (ChileFG16CP), when in reality ‘*every person is very different in themselves regardless of nationality*’ (ChileFGTCP). The idea of broad cultural categories was problematised, as different cultures can be found in the same country (ChileFGTCP) or even the same city (ChileFG1CP), and cultural groups were not seen as homogeneous: ‘*There are Colombian families who think very differently (from others)*’ (ChileFG11CP).

In the context of such diversity, the necessity of abstract cultural knowledge was critiqued, with one participant suggesting that ‘*maybe it is not even necessary to contact institutions or outsiders in the country, but to learn from the person themselves how the subject is approached*’ (ChileFG11CP).

Chilean participants saw potential in the toolkit for ‘*Integrating the intersubjectivity of each person*’ by providing ‘*a meeting point for dialogue and co-construction*’ (ChileS9CP). It was emphasised that, in the context of diversity, ‘*intersubjectivity is something we always need to address*’ (ChileFG16CP).

Relatedly United Kingdom participants stressed the value of co-production of mental health services and treatments: one participant spoke of how the process of formulating what is best for a service user ‘*should be an exploration process of you together*’ (UKFG12ST), while another suggested that the toolkit should be used in a way that incorporates ‘*experts-by-experience*’ for ‘*what they might bring to a conversation about ethical clinical psychology practice*’ (UKS2GW). It was proposed that the toolkit should be improved using service user feedback ‘*since their ideas will have a huge impact*’ (UKFG1SW).

Both Chilean and United Kingdom participants stressed the importance of clinicians’ awareness of their own cultural background and its influences, with one suggesting that the toolkit should ‘*incorporate more explicitly the need to problematise the therapist’s stance towards his or her own culture*’ (ChileS5CP).

These differing emphases may relate to the professional composition of the samples and the particular service and sociocultural contexts in which participants were working. They also illustrate the value of a toolkit that can accommodate contextual variation. More detailed signposting could help users consider both their own assumptions and the perspectives of others when reflecting on intercultural mental healthcare.

### Theme 3: practical guide, or space to reflect?

#### Where the toolkit ‘fits’ into practice

Some participants considered ‘*where the toolkit was intended to fit in practice*’ (UKS3CP) and asked whether it would be used by individual practitioners or at the level of policy change (UKFG2TCP).

Participants from both groups saw clinical supervision as an appropriate place for using the toolkit, as ‘*an important mechanism for considering ethical tensions in practice*’ (UKS2GW). They felt that supervision and peer supervision were missing from the model (ChileFG6CP) and saw a need to include them more explicitly (ChileFG6CP, UKS2GW).

Participants also thought the toolkit ‘*would be good in multidisciplinary settings*’ (UKFG4CP) and could ‘*foster collaboration and communication among professionals from different disciplines*’ (UKS6CP), which could help clinicians think differently about cases (ChileFG1CP), although one participant thought the psychological language of the toolkit such as ‘*formulation*’ might need explanation to professionals of other disciplines (UKS6CP).

Both groups of participants also stressed that ‘*Conversations need to happen upwards*’ (UKFG5AC) with managers and policymakers for the toolkit to be properly supported in practice:

It has to go all the way up too – it has to go up so that the policies are also incorporated … they say “you have to work on this” … but in the end all this falls on the professional practice of the one who is there on the front line (ChileFG2CP)

Participants raised issues with *‘systemic issues on referral pathways’* (UKFG8AC), including the gatekeeping of services by GPs (UKFG5AC) and the siloing of specialised services that do not work well together (ChileFGCP). Participants also identified a lack of diversity among practitioners (UKFG7PS) and systemic racism (UKS4SW) as important issues. They questioned how they could best use the toolkit to ‘*advocate for systemic changes rather than only used on an individual level*’ (UKFG8AC) and how to introduce it to other institutions, such as care networks and courts (ChileFG28CP). One participant suggested that systemic issues should be addressed prior to the other stages in the toolkit journey model (UKFG8AC).

Participants from both groups saw a need for the toolkit, describing it as valuable, relevant, and useful. One Chilean participant spoke of ‘*how wonderful it would have been*’ to have had the toolkit when dealing with previous ethical challenges (ChileFG2CP), and a United Kingdom participant said the toolkit ‘*seems extremely valuable … for navigating what can often be a very bewildering process of coming to understand blind spots and gaps in knowledge, unknown unknowns*’ (UKS4SW).

#### United kingdom participants emphasise practicality and need for guidance

While the toolkit has proved a source of support for some, others identified challenges. Some United Kingdom participants reported that they found the toolkit ‘confusing to apply’ (UKS2GW) and were unsure whether it was for ‘reflection’ or ‘a practical tool’ (UKFG3PHW). One participant found it unclear ‘how the model was approaching ethical deliberation or reflection’ beyond highlighting ethical challenges. The concern was that, in their view:

It doesn’t provide those applying it with a way to navigate the balancing or trade-offs that will inevitably arise. (UKS2TCP)

United Kingdom participants also suggested that the toolkit ‘*could be simplified a lot*’ or narrowed down ‘*to some key points*’ (UKS3CP). A need was seen for the toolkit to be ‘*simple to simplify the complexity*’ (UKFG1CW), to provide ‘*more hands-on guidance*’ (UKS12AC), and to give specific information about what should be discussed (UKS3CP).

Student participants thought that the toolkit’s function could be clarified with more training resources (UKS13ST, UKS14ST), including ‘*videos, interactive activities, etc*.’ (UKS13ST), and pointed out that, especially in a global context, the model might be interpreted differently by different people (UKFG12ST).

#### A toolkit for ethical reflection

Chilean participants opposed simplification because ‘*there is a risk in simplifying the complex*’ (ChileFGCP). They did not think the model should provide too much rigid guidance:

I don’t think that you should have a ‘manual’ or a ‘recipe book’ of what to do, how to do it etc. I think that’s dangerous (ChileFG10CP)

Participants problematised institutions where ethical considerations are ‘relegated to a committee’ (ChileFG11), proposing instead that the ethical ‘dimension’ should always be taken into account (ChileFG10CP). They thought that the toolkit could be beneficial by providing ‘a space for conversation and discussion.’ (ChileFG8CP) that ‘promotes the visibility of the ethical dimension’ (ChileS10CP) while organising aspects of a case for reflection:

I think it’s an interesting idea to have a tool – not to simplify the complex, but to order it a little, and ordering it enables you to bring other concepts to bear, other visions of the world. (ChileFG9CP)

Participants cautioned against the toolkit becoming another ‘*checklist*’ (ChileFG17, ChileFG9CP), or ‘*a guide or a manual that one has to complete*’ (ChileFGTCP). They thought it should not be used to make decisions ‘*on autopilot, where we lose that ethical dimension*’ (ChileFG12CP), and stressed the importance of using it to reflect on the fact that decisions have serious implications for service users’ lives (ChileFG2CP). Rather than a checklist or a ‘*list of intervention objectives*’ (ChileFG2CP), participants saw value in the toolkit’s ethical process:

The question is not whether we are going to provide all the solutions. It’s more about whether we are going to have an ethical pathway or not. (ChileFG6CP)

Some United Kingdom participants also cautioned against using the toolkit ‘*like a checklist*’ (UKS14ST), and positively emphasised the reflective potential of the toolkit, considering it ‘*extremely valuable as a way of exploring ethical issues before and as they arise*’ (UKS4SW) while posing important questions for practitioners to reflect on (UKFG9SW).

## Discussion

### Commonality in intercultural ethical dilemmas across two cultural contexts

In using ETHICA-4P in two diverse contexts (Chile and the United Kingdom), participants identified ethical dilemmas that were common in intercultural mental health care in both places and that have been recognised in other studies such as language and communication issues [12,48]; more hidden barriers, such as the way in which emotional concepts may vary between languages and cultures [49,50]; and how these may create further difficulties when interpreters are involved, as nuances may be lost or distorted [12,51,52]. The analysis also identified ethical dilemmas relating to gender norms, family roles and violence, consistent with research showing that migrant and refugee women face higher risks of gender-based violence, particularly if isolated or undocumented [53,54]; and that patriarchal practices and attitudes towards child discipline present ethical tensions for practitioners in navigating safeguarding within cultural contexts [55,56]. Differences in explanatory frameworks for mental health and expectations of clinical relationships were also identified as key ethical challenges, consistent with literature on cultural variations in understandings of distress [14,57] including how this may lead to service disengagement [58,59].

Triangulation of our findings with external studies provides further support for the utility of the ETHICA reflective process in drawing out relevant risks and considerations. If practitioners in different contexts encounter similar ethical challenges, a tool that fosters shared reflection and solutions could be valuable. Perhaps even more importantly, although it was beyond the scope of these workshops, it was acknowledged that the toolkit is designed with both a problem analysis and a ‘solution seeking’ feature and that each of the ethical dilemmas identified could therefore be addressed through further consideration of Place, People, Principles and Precedents at each stage on the clinical journey.

### Place-based approaches to interculturality and implications for the toolkit

United Kingdom participants advocated for increased practitioner knowledge of specific cultures and culturally adapted interventions. This aligns with models of cultural competence that emphasise cultural knowledge [34,60]. However, this approach has been critiqued: it may not be feasible to expect practitioners to master all relevant cultural knowledge in diverse contexts [61], and risks essentialising cultures and ‘othering’ service users [62,63]. Chilean participants’ emphasis on diversity within cultural groups and concerns regarding stereotyping reflect these critiques.

Calls for culturally adapting interventions raised by United Kingdom participants are well supported in the literature [64,65]. Culturally adapted CBT, for example, has been identified as a human rights priority [66]. However, designing practical guidance for such adaptations is complex, given the need to tailor both surface and deep structure elements [67].

Within the Chilean sample, participants more frequently framed ethical dilemmas in relation to structural inequalities and migration trajectories. This resonates with literature on structural vulnerability and its links to mental health [68–70], as well as with structural competency approaches [71]. Chile’s tradition of community psychiatry [72] provides one potentially relevant contextual consideration, although the present study cannot determine whether this or other contextual or sample characteristics account for the pattern observed. In this sample, United Kingdom participants more frequently discussed cultural knowledge and adaptation, whereas Chilean participants more frequently foregrounded structural aspects of migration. Chileans’ suggestions to include legal knowledge in the toolkit mirror their structural orientation. However, this raises challenges about scope: can a toolkit provide relevant legal or cultural ‘facts’ for all contexts? It may be more practical to use the toolkit to signpost practitioners more clearly towards identifying local sources of such Principles and Precedents, including legal knowledge.

Both groups valued co-construction and intersubjectivity, proposing that meaning-making in therapy should be a collaborative process. This aligns with social constructionist approaches, which view knowledge as negotiated through interaction [73,74]. Co-construction has been suggested as a natural extension of person-centred and intercultural care [23], and resonates with models of cultural humility, which prioritise openness, reflexivity, and awareness of one’s own biases [75,76]. Integrating cultural and structural humility into cultural competence frameworks has been recommended [71,77].

Participants’ advocacy for co-production – involving service users in ethical decision-making – is consistent with best practices in inclusive service design [77,78] and with the principles underlying the toolkit’s development. Future work should further prioritise the inclusion of experts-by-experience in refining the toolkit.

### Decision-making with the toolkit

Participants agreed that clinical supervision could provide a natural setting for using the toolkit. Supervision is recognised as key to ethical practice and professional development [79,80]. Embedding the toolkit in supervision or multidisciplinary settings could enhance reflective practice and collaborative ethical decision-making [81].

Participants highlighted the need for the toolkit to address systemic barriers and involve not just frontline practitioners, but also managers and policymakers. This aligns with research identifying systemic issues as key obstacles to equitable mental healthcare [4,10].

United Kingdom participants sought more structured guidance in the toolkit, echoing tools such as the Social GRRAAACCEEESS framework [82], designed to aid reflection on social differences. However, caution is warranted: reducing ethics to checklists risks reifying categories and missing the dynamic, contextual nature of cultural identities [83,84]. Participants voiced similar concerns regarding oversimplification.

Others valued the toolkit’s role in organising complex information, encouraging reflection, and maintaining ethical awareness in uncertainty. This approach mirrors Pope’s vision of ethics as a creative, iterative process [18]. It is also consistent with Ilgen et al.’s findings that embracing uncertainty enhances complex clinical decision-making [85]. As one participant put it:

The question is not whether we are going to provide all the solutions. It’s more about whether we are going to have an ethical pathway or not. (ChileFG6CP)

Taken together, these findings highlight the importance of the flexibility and adaptability that was designed into the toolkit. Each group was able to access both analytical axes of ETHICA-4P – the clinical journey and the 4P’s – in ways that were meaningful and relevant to their unique context.

ETHICA-4P was intended as a tool from which users could draw upon relevant elements to simplify complex challenges. Participants in this study demonstrated different approaches to using the toolkit (for example, foregrounding systemic versus individual-level analysis). These patterns may be understood in relation to the place-specific needs and circumstances of the two study contexts, while also potentially reflecting differences in professional composition and data collection. The resulting points of convergence (e.g. shared ethical challenges) and divergence (e.g. different areas being foregrounded when considering potential responses) illustrate the value of retaining flexibility within the toolkit. These findings suggest that, rather than adding more content to the toolkit – as inevitably this will be context-locked – clearer signposting to the toolkit website and its resources may support users who are approaching the toolkit with different needs.

### Strengths and limitations

A strength of this exploratory study is the inclusion of perspectives from two distinct service contexts and, in the United Kingdom, a breadth of professional backgrounds. Limitations include the small purposive, convenience and snowball samples, different recruitment and data-collection procedures across settings, and differing professional composition: the Chilean sample comprised psychologists and trainee psychologists, whereas the United Kingdom sample was multi-professional. These features limit direct comparison and the transferability of findings. Contrasts between the two groups should therefore be understood as contextual observations within these samples rather than as country-level effects. Translation may have reduced linguistic nuance, and the use of minuted rather than verbatim United Kingdom focus-group records introduced an additional layer of interpretation. Service-user perspectives were absent and should be prioritised in future work. As an early beta test, the study also does not establish whether ETHICA-4P improves clinical decision-making, patient outcomes or service quality; further evaluation is required.

Researcher influence also requires consideration because members of the ETHICA-4P development team were involved in data collection and later interpretation. Using structured prompts and a postgraduate researcher to conduct the primary analysis introduced some analytical distance, but codes and themes were subsequently discussed with the wider team, including toolkit developers. Researcher subjectivity was therefore treated as part of the interpretive process rather than as something that could be eliminated; reflexive team discussion was used to examine and refine developing interpretations.

## Conclusion

This study offers exploratory insights into developing ETHICA-4P as a resource for navigating intercultural ethical dilemmas in mental healthcare. Shared challenges across the two participant groups suggest value in tools that support reflective, equitable practice, while differing emphases across contextual accounts indicate a need to clarify the toolkit’s scope, epistemology and intended use. Integrating service-user input and strengthening attention to language, structural issues, supervision and co-production are priorities for future development. ETHICA-4P may be most useful as a flexible space for reflective ethical decisions rather than as prescriptive guidance.

## Supplementary Material

Supplemental Material

## Data Availability

The data that support the findings of this study are available from the corresponding author upon reasonable request. Due to confidentiality agreements and the sensitive nature of focus group data involving human participants, transcripts and survey responses are not publicly available.
